# Evaluation of the Effect of Different Inhalation Agents on Ovaries with Hyperthermic Intraperitoneal Chemotherapy: An Experimental Study

**DOI:** 10.3390/medicina60091403

**Published:** 2024-08-27

**Authors:** Özlem Şen, Esra Aslan, Dilek Kalaycı, Ayşegül Küçük, Semih Başkan, Şaban Cem Sezen, Mustafa Arslan, Yusuf Ünal, Murat Tosun

**Affiliations:** 1 Department of Anesthesiology and Reanimation, Trabzon Kanuni Training and Research Hospital, Trabzon 61250, Turkey; turanozlem1@yahoo.com.tr; 2 Department of Histology and Embryology, Faculty of Medicine, Afyonkarahisar Health Sciences University, Afyonkarahisar 030030, Turkey; esra.aslan@afsu.edu.tr (E.A.); drmtosun@yahoo.com (M.T.); 3 Department of Anesthesiology and Reanimation, Ankara A.Y. Oncology Training and Research Hospital, Ankara 06200, Turkey; drdkalayci@hotmail.com; 4 Department of Physiology, Faculty of Medicine, Kutahya Health Sciences University, Kütahya 43110, Turkey; kucukaysegul@hotmail.com; 5 Department of Anesthesiology and Reanimation, Faculty of Medicine, Ankara Yıldırım Beyazit University, Ankara 06800, Turkey; drsemkan@yahoo.com; 6 Department of Histology and Embryology, Faculty of Medicine, Kırıkkale University, Kırıkkale 71450, Turkey; sezenscem@gmail.com; 7 Department of Anesthesiology and Reanimation, Faculty of Medicine, Gazi University, Ankara 06560, Turkey; yunal71@yahoo.com

**Keywords:** HIPEC, sevoflurane, desflurane, ovary, cisplatin, caspase 3, p53

## Abstract

*Background and Objectives*: Cisplatin is a chemotherapeutic drug that is frequently used with hyperthermic intraperitoneal chemotherapy (HIPEC). Cisplatin-induced gonadotoxicity leads to a depletion of the ovarian reserve, causing premature ovarian insufficiency. This study aimed to investigate the impact of hyperthermia on cisplatin-induced ovarian toxicity and to determine whether sevoflurane or desflurane could be a more appropriate choice of anesthetic for reducing ovarian toxicity in HIPEC procedures. *Materials and Methods*: A total of 24 New Zealand rabbits were randomly divided into 4 groups as follows: Group H: HIPEC (cisplatin 7 mg/kg), Group HS: HIPEC (cisplatin 7 mg/kg) + 3% sevoflurane (2 h), Group HD: HIPEC (cisplatin 7 mg/kg) + 6% desflurane (2 h), and Group C: Control (Saline). Two catheters were placed in the abdominal cavity, the upper and lower quadrants. The perfusate was heated to 42 °C and given intraperitoneally for 90 min at a rate of 4 mL/min by catheters. Ovarian tissues were collected for Hematoxylin and Eosin staining and immunohistochemical analysis. *Results*: The primary follicle number was significantly decreased in Group H and HD compared to the C group (*p* < 0.05). Bax expression was high in Group H, according to all groups (*p* < 0.0001). Bax expression significantly decreased after sevoflurane, compared to group H (*p* = 0.012). The bcl-2 expression decreased in all groups compared to the C group. Bcl-2 expression was increased with sevoflurane compared to the H group (*p* = 0.001). Caspase 3 and p53 expression increased in all groups compared to the C group. p53 expression was decreased with sevoflurane and desflurane compared to the H group (*p* = 0.002, *p* = 0.008, respectively). *Conclusions*: The application of cisplatin with the intraoperative HIPEC method caused ovarian damage. According to our results, sevoflurane anesthesia could be a better option in mitigating cell death I the n ovarian reserve (follicle count) and apoptosis in the HIPEC procedures. We think that our findings should be supported by large series of clinical and experimental studies.

## 1. Introduction-Objective

Hyperthermic intraperitoneal chemotherapy (HIPEC) is a type of locoregional chemotherapy which is used combined with cytoreductive surgery for specific types of cancer affecting the peritoneum [1]. The effectiveness of an optimal combination of cytoreductive surgery and HIPEC has been proven in the treatment of peritoneal carcinomatosis of different primary origins, especially in the absence of hematogenous metastases [2]. Thanks to HIPEC, high concentrations of chemotherapeutic drugs can be used in targeted delivery to the abdominal cavity with minimal systemic effects and bypassing blood–peritoneal barriers. HIPEC provides an additional therapeutic modality. Hyperthermia also induces apoptosis, inhibits angiogenesis, and has a direct cytotoxic effect by promoting protein denaturation [3]. Kong et al. showed that SKOV-3 cells can resist hyperthermia-induced apoptosis via HSP27, which promotes bcl-2 expression and inhibits Bax and caspase-3 expression [4]. Preclinical data suggest that hyperthermia at temperatures up to 42 °C may have a synergistic effect with certain (but not all) chemotherapy agents, including cisplatin and paclitaxel [5,6]. Long-term survival and quality of life in patients with various cancers increased with continuous improvements in chemotherapeutic treatments and combined chemotherapeutic procedures. Chemotherapeutic procedures used in the reproductive age group can lead to long-term complications such as premature ovarian failure and infertility in women. Cisplatin is frequently used as a chemotherapeutic drug in HIPEC. Cisplatin (Cis-diamminedichloroplatinum) is an alkylating chemotherapeutic agent used in sarcomas, ovarian germ cell tumors, and many solid tumors. Cisplatin exerts its anticancer effect by interfering with DNA synthesis and generating reactive oxygen radicals. Nephrotoxicity, hepatotoxicity, myelosuppression, and genotoxicity are the primary limitations for the clinical use of cisplatin as an antitumor medication [7].

Cisplatin-induced gonadotoxicity leads to depletion of ovarian reserve in women of reproductive age, causing premature ovarian insufficiency. Therefore, investigating the molecular mechanisms that regulate cisplatin-induced ovarian damage will be crucial in developing agents to preserve or restore ovarian functions in many young women who have survived cancer after chemotherapy [7]. The molecular mechanism of cisplatin toxicity is associated with the sequential thermal exchange of hydroxyl ligands with two chloride groups, leading to its biological activation. After activation, cisplatin acts as a potent nucleophilic agent, attacking DNA targets and causing the formation of inter/intrastrand cross-links, protein adducts, and DNA strand breaks, resulting in inhibition of replication, transcription, and cell deaths. Additionally, oxidative stress is another mechanism that plays a significant role in genotoxicity. Furthermore, cisplatin triggers cellular stress that can induce apoptosis in a caspase-dependent manner [8].

Researchers have attempted to develop protective adjuvants such as antioxidant reactants to overcome these side effects and elucidate their protective mechanisms [9,10].

An increasing number of cancer patients undergo multiple surgical procedures and receive anesthesia before, during, or immediately after surgery, potentially affecting their anticancer drug regimen. The potential risk of toxicity caused by the interaction between drugs used in anesthesia and cancer treatment remains a concern in HIPEC procedures, which involve the intraoperative use of anticancer drugs and have emerged as a new clinical oncology field for cancer treatment. The selection of appropriate anesthesia is of great importance during cancer treatment, as some anesthetics can have a significant impact on the metabolism and cause increased toxic reactions of cytostatic or cytotoxic drugs [11]. Anesthetic gases used in general anesthesia have recently become interesting due to potential mutagenic/genotoxic effects among exogenous compounds [12]. Sevoflurane and desflurane are inhalation anesthetics commonly used in HIPEC applications. Sevoflurane (2,2,2-trifluoro-1-[trifluoromethyl] ethyl fluoromethyl ether) and desflurane (2,2,2-trifluoro-1-fluoroethyl-difluoromethyl ether) are very common volatile anesthetics used for general anesthesia. About 5% of inhaled sevoflurane is metabolized by oxidative metabolism in liver cells, and a minor percentage is defluorinated by cytochrome P-450 in the kidney. In addition, it metabolizes far less to F^−^ compounds than sevoflurane. [13] Desflurane acts the most rapidly among volatile anesthetics, due to its slow solubility in blood, which is related to the lowest blood–gas partition coefficient [14]. Experimental animal studies on the effects of volatile anesthetics on oxidative stress are limited in the literature [15]. Some in vivo and in vitro studies have shown that sevoflurane potentially induces lipid peroxidation [16]. A study evaluating different tissues such as the liver, kidney, and brain of rats exposed to sevoflurane and desflurane have reported that oxidative and antioxidative activities varied depending on the types of tissue [17].

This study aimed to investigate the impact of hyperthermia on cisplatin-induced ovarian toxicity and to determine whether sevoflurane or desflurane could be a more appropriate choice of anesthetic for reducing ovarian toxicity in HIPEC procedures.

## 2. Materials and Method

### 2.1. Experimental Animals

Ethical approval was obtained from Gazi University Ethical Committee of Experimental Animals (G.U.ET—16.005); the experiment was conducted in the Gazi University Animal Laboratory, and carried out in accordance with ARRIVE guidelines. All laboratory procedures were in accordance with the Guide for the Care and Use of Laboratory Animals in Ankara, Turkey. In the current study, 24 New Zealand breed rabbits weighing between 1500 and 2000 g were used, under equal environmental conditions. The rabbits were fed until 2 h before drug administration, the temperature was kept at 20–21 °C and a 12 h day–night period was provided. A sample size/power analysis could not be performed due to the restriction imposed by the animal research committee on the number of animals allowed. Consequently, the number of rabbits in each group was determined based on the committee’s authorization.

One author (M.A.) was aware of the grouping, conduct of the trial, and outcome assessment. Histological evaluation and data analysis were conducted by investigators blinded to this study.

### 2.2. HIPEC Model Creation

**Figure 1:**
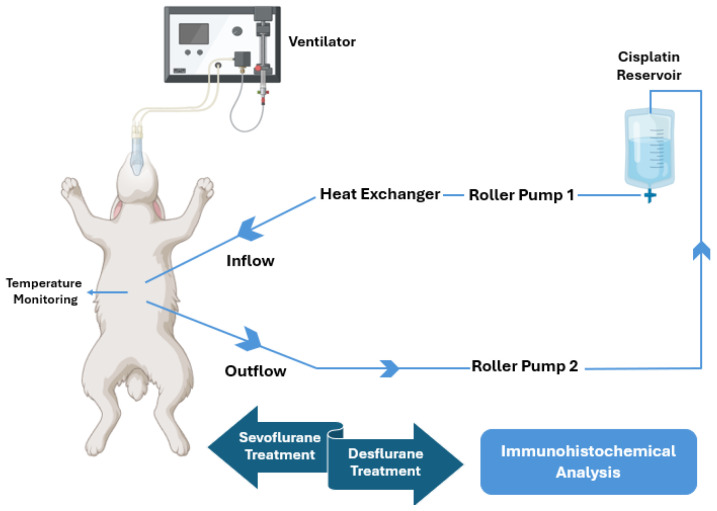
Schematic representation of the experimental HIPEC circuit.

All rabbits were anesthetized using intraperitoneal 80 mg/kg ketamine (Ketalar; Pfizer PFE İlaçları, İstanbul, Turkey); a suitable laryngeal mask was applied, and they were connected to a ventilator. In all rabbits, two catheters were placed in the abdominal cavity, one in the upper quadrant and one in the lower quadrant. The catheters were connected to a closed perfusion system containing 1 L of physiological solution. The perfusate was heated to 42 °C and administered to the intraperitoneal cavity at a rate of 4 mL/min for 90 min (Figure 1).

The rabbits were randomly divided into 4 groups, with 6 rabbits in each group.

Group H: HIPEC (Cisplatin 7 mg/kg)
Group HS: HIPEC (Cisplatin 7 mg/kg) + 3% Sevoflurane (2 h)
Group HD: HIPEC (Cisplatin 7 mg/kg) + 6% Desflurane (2 h)
Group C: Control Group (Saline)

In Group H, cisplatin 7 mg/kg (Cisplatin Hospira 100 mg/100 mL ONCO-TAIN, Hospira, Warwickshire, UK) was applied using the HIPEC method. In Group HS, in addition to cisplatin, 3% sevoflurane was administered for 2 h using the HIPEC method. In Group HD, in addition to cisplatin, 6% desflurane was administered for 2 h using the HIPEC method. In Group C, only intraperitoneal physiological saline was applied and hyperthermia was applied.

After a 24 h period, tissue samples were obtained from the sacrificed rabbits and fixed in 10% formalin. After routine histological tissue processing, the tissues were embedded in paraffin. Sections of 5 μm thickness from these blocks were taken on Poly-L-Lysine and conventional slides. Classical slides were stained with Hematoxylin-Eosin (HE), and Poly-L-Lysine slides were stained immunohistochemically with p53, Bax, bcl-2, and caspase-3. An evaluation was performed at the molecular level.

### 2.3. Immunohistochemical Staining

The sections were rehydrated by passing through alcohols with decreasing concentrations (room temperature for 20 min). After antigen retrieval with citrate buffer solution (pH = 6.0) in a microwave, the sections were treated with 3% methanol and H_2_O_2_ to block endogenous peroxidase activity. To prevent background staining, the sections were treated with protein blocking solution for 5 min. Subsequently, the sections were incubated overnight at +4 °C with primary antibodies, including caspase-3 (ab 4051, 1/100, Abcam, Cambridge, UK), Bax (sc-526, 1/100, Santa Cruz Biotechnology, Dallas, TX, USA), Bcl-2 (PA5-27094, 1/100, Thermofisher Scientific, Rockford, IL, USA), and p53 (sc-6243, 1/100, Santa Cruz Biotechnology, Dallas, TX, USA). The following day, Ultra Vision Detection System Large Volume Anti-Polyvalent, HRP (RTU) (Thermofisher Scientific, Fremont, CA, USA) kit was applied as the secondary antibody. AEC chromogen was used for staining, and Mayer’s Hematoxylin (Thermofisher Scientific) was used for counterstaining. The sections were then mounted using an aqueous mounting solution and examined under a light microscope. The Nikon DS 4.2 Image Analysis software was used during the examination. Immunoreactivity was evaluated under a light microscope, and cytoplasmic staining was considered positive.

### 2.4. Image Analysis

The obtained preparations were evaluated by an image analysis program (NIS Elements [Nikon, Tokyo, Japan]). Primordial, primary, secondary, and tertiary follicules were counted in the entire ovarian area using Hematoxylin–Eosin staining. In the evaluation of immunohistochemical staining, stromal cells with immunopositive staining were counted in 5 different areas in each section under ×20 objective magnification.

### 2.5. Statistical Analysis

Statistical analysis was performed with the Statistical Package for Social Sciences (SPSS 20.0, Chicago, IL, USA) software package. The Shapiro–Wilk test was used for comparisons of the variable groups. One-Way Analysis of Variance (ANOVA) and Tukey’s post hoc tests were performed to assess the results. All data were expressed as mean ± standard deviation (SD) values. A value of *p* < 0.05 was considered statistically significant.

## 3. Results

### 3.1. Histopathological Findings

The number of primordial follicles was counted using light microscopy; this significantly decreased in the H and HD groups compared to the C group (*p* < 0.0001, *p* < 0.0001, respectively), and significantly increased in the HS group compared to the H group (*p* = 0.003). Additionally, the number of primordial follicles significantly decreased in the HD group compared to the HS group (*p* = 0.019) (Table 1). Similarly, the number of primary follicles significantly decreased in the H and HD groups compared to the C group (*p* = 0.001, *p* = 0.005, respectively), and significantly increased in the HS group compared to the H group (*p* = 0.013) (Table 1). In addition, the number of secondary follicles significantly decreased in the H group compared to the C group (*p* = 0.031) (Table 1). The number of tertiary follicles was significantly higher in the HS group compared to the C and H groups (*p* = 0.007, *p* = 0.004, respectively) (Table 1).

### 3.2. Immunohistochemical Findings

**Figure 2:**
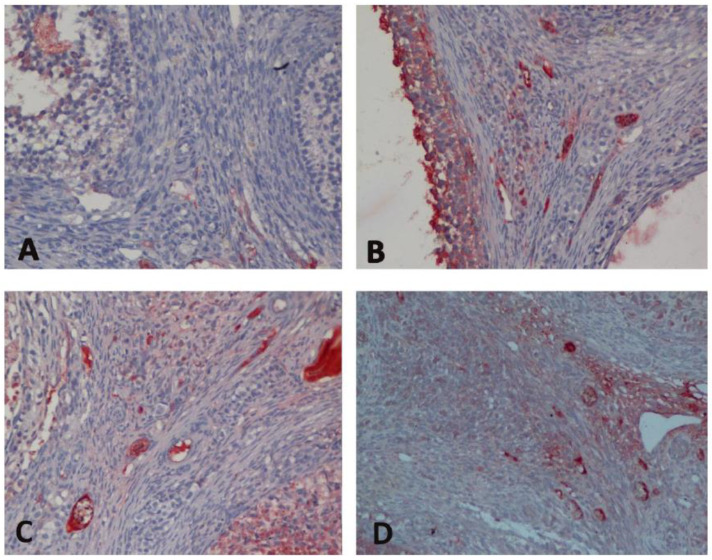
Immunohistochemical images of Bax staining in the study groups ((**A**); Group C, (**B**); Group H, (**C**); Group HS, (**D**); Group HD). Bax, B-cell lymphoma 2-associated X protein.

**Figure 3:**
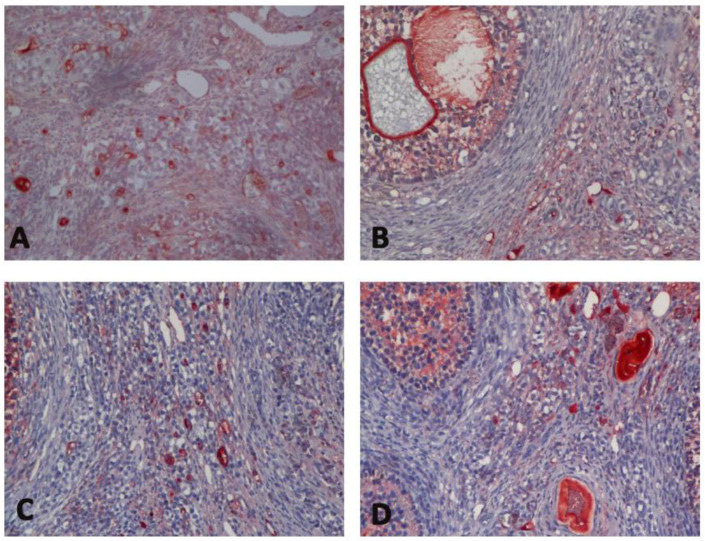
Immunohistochemical images of bcl-2 staining in the study groups ((**A**); Group C, (**B**); Group H, (**C**); Group HS, (**D**); Group HD). Bcl-2, B-cell lymphoma 2.

**Figure 4:**
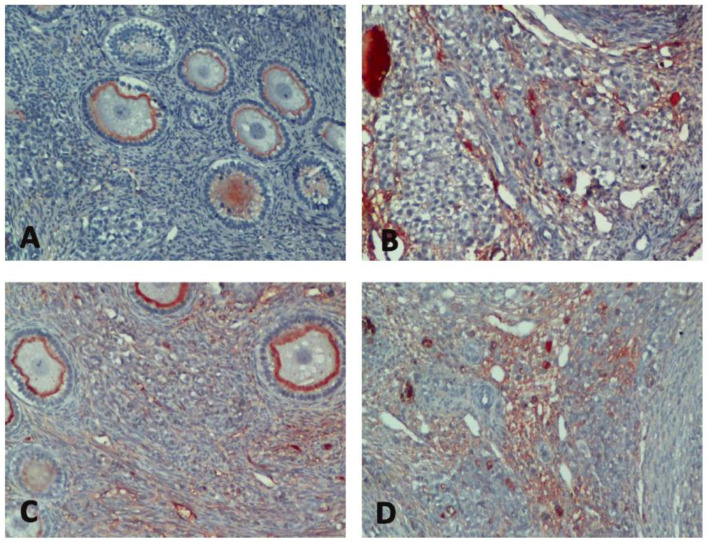
Immunohistochemical images of caspase 3 staining in the study groups ((**A**); Group C, (**B**); Group H, (**C**); Group HS, (**D**); Group HD).

**Figure 5:**
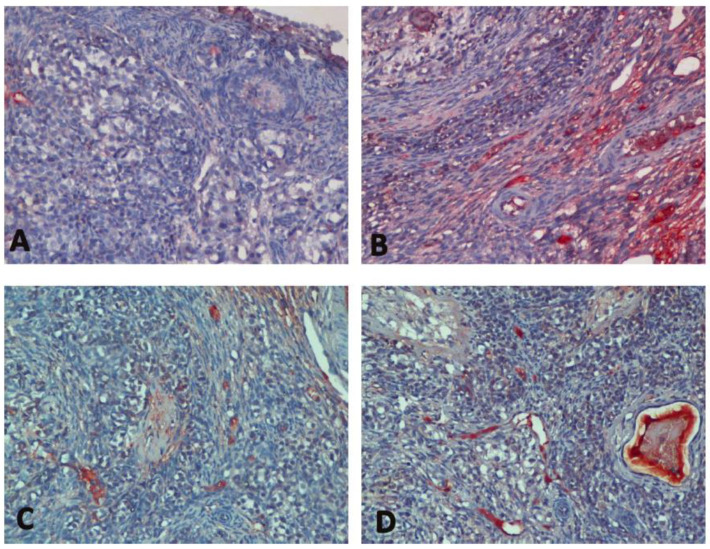
Immunohistochemical images of p53 staining in the study groups ((**A**); Group C, (**B**); Group H, (**C**); Group HS, (**D**); Group HD).

In immunohistochemical evaluation, it was determined that the increase in Bax expression was highest in Group H and there was a significant difference between the C group and all groups (*p* < 0.0001) (Table 1, Figure 2). Bax expression reduced significantly with sevoflurane (*p* = 0.012). In addition, a decrease of bcl-2 expression was observed in all groups compared to the C group (*p* < 0.0001, *p* = 0.032, *p* < 0.0001, respectively) (Table 1, Figure 3). Bcl-2 expression significantly increased with sevoflurane compared to Group H (*p* < 0.0001) (Table 1, Figure 2). There was a significant increase in caspase 3 expression in all groups compared to the C group (*p* < 0.0001) (Table 1, Figure 4). Similarly, there was a significant increase in p53 expression in all groups compared to the C group (*p* < 0.0001, *p* = 0.003, *p* < 0.0001, respectively). p53 expression significantly reduced with both sevoflurane and desflurane compared to Group H (*p* = 0.002, *p* = 0.008, respectively) (Table 1, Figure 5).

## 4. Discussion

The pharmacology and clinical pharmacodynamics of chemotherapeutic drugs and their interactions with anesthetics are often poorly understood. There is also a lack of information regarding the combined effects of anesthetics and cytotoxic drugs on both healthy and tumor cells. Since chemotherapeutic drugs are not selective towards tumor cells, they can also damage healthy cells, emphasizing the importance of anesthesia selection for a surgical procedure. However, an anesthesiologist should be aware of the potential side effects of each administered chemotherapy drug, along with common adverse reactions such as allergic reactions, nausea, vomiting, or flushing [18].

The potential risk of toxicity caused by interactions between anesthesia and chemotherapeutics is considered a negative aspect of HIPEC procedures. The aim of the current study was to investigate the effect of hyperthermia on cisplatin-induced ovarian toxicity and to investigate whether sevoflurane or desflurane is a more appropriate anesthetic option to reduce ovarian toxicity with HIPEC procedures. The results of this study showed that hyperthermia alone did not cause adverse outcomes in the C group. In terms of ovarian reserve (follicle count), a significant decrease in both primordial and growing follicle numbers was observed with hyperthermic cisplatin application. In the group treated with simultaneous desflurane during HIPEC, there was a decrease in primordial and primary follicle numbers compared to the C group, while there was no significant change in their secondary and tertiary follicle numbers. Sevoflurane prevented cisplatin-induced follicular loss. The numbers of primordial, primary, and tertiary follicles were significantly higher in the C group compared to groups receiving hyperthermia + cisplatin. These results suggest that sevoflurane anesthesia may provide a protective effect for the ovaries in cisplatin-induced ovarian toxicity during HIPEC.

It is known that cisplatin, a chemotherapeutic agent, causes damage to ovaries by reducing primordial follicles, and also decreases anti-Müllerian hormone (AMH) levels and increases ovarian damage [19]. Cisplatin is categorized as a member of the intermediate gonadal-risk group of drugs [20]. Chemotherapy has been reported to trigger early ovarian insufficiency (POI) and infertility by depleting the primitive follicle pool in young female cancer patients [21]. Since chemotherapy accelerates ovarian follicle loss and leads to infertility, quantitative measurement of follicles is the most effective way to assess fertility status in women. This study found that both dormant primordial and growing follicles were impacted by cisplatin treatment, leading to more extensive ovarian damage [22]. There is evidence that exposure to cisplatin in mouse and rat ovaries causes loss of ovarian reserve and an increase in follicular atresia. This demonstrates the particular sensitivity of immature oocytes [23,24].

Taskin et al. investigated the efficacy of sildenafil combined with cisplatin treatment, which was due to its antioxidant and antiapoptotic effects in protecting against ovarian toxicity. They observed a significant decrease in numbers of primordial, secondary, and tertiary follicles after cisplatin treatment, and when sildenafil was added to cisplatin they found an improvement in the number of primordial follicles but no improvement in the number of secondary and tertiary follicles. The number of primary follicles did not differ between the groups [7]. In our study, sevoflurane anesthesia increased the numbers of primordial and primary follicles. The number of secondary follicles was only low in the H group. Additionally, we did not observe a significant decrease in the number of tertiary follicles, even in the H group. We found a significantly high number of tertiary follicles in the HS group compared to the C group.

One of the targets of chemotherapeutic agents is the granulosa cells that play a key role in follicle formation and provide support for oogenesis. Damage to granulosa cells during chemotherapy can negatively affect oocyte maturation and lead to follicular destruction. Therefore, follicular damage is considered the main cause of ovarian insufficiency and infertility caused by chemotherapy [25]. Yücebilgin et al. demonstrated the cytotoxic effects of cisplatin on primordial follicles, which constitute a large part of the ovarian reserve [26]. Consistent with the literature, we found a reduction in primordial follicles due to cisplatin, but did not observe a significant decrease in the number of tertiary follicles. There are studies that indicate the importance or lack of importance of tertiary or antral follicles in the ovarian reserve [27]. A study found that Caffeic acid phenethyl ester (CAPE) contributed positively to the number of tertiary follicles, but this contribution was not reflected in AMH levels. CAPE is one of the most active compounds of propolis obtained from honey. It has antioxidant, anti-inflammatory and anticarcinogenic characteristics, and regulates apoptosis. AMH is considered as the best indicator of ovarian reserve [19].

Anesthetic drugs that cause less oxidative stress and have more antioxidant properties are a better choice, especially in critically ill patients who are already under increased oxidative stress, such as after surgery. Animal studies on volatile anesthetics and tissue oxidative stress are limited in the literature. Sato et al. found that sevoflurane has the potential to induce in vivo and in vitro lipid peroxidation [16]. Another study reported that desflurane induced local and systemic oxidative stress in pig lungs, while sevoflurane exhibited antioxidant properties [28].

One of the mechanisms of ovarian damage caused by cisplatin is a much more complex condition which involves the interaction of inflammation and cell deaths. Cytotoxic chemotherapy and radiotherapy regimens deplete follicle reserve and cause POI by inducing apoptosis in oocytes and the surrounding granulosa cells [29]. The mechanism that leads to ovarian damage involves the activation of apoptotic pathways through the p53 signaling family, DNA damage caused by oxidative stress, and the production of free radicals. Exposure to cisplatin increases DNA damage in ovarian cells and results in apoptosis, as evidenced by the activation of apoptotic genes such as caspase 3 [9]. It has been reported that the intrinsic pathway of apoptosis plays a crucial role in the pathogenesis of premature ovarian failure. The process begins with an increase in the permeability of the mitochondrial membrane, primarily caused by chemotherapy-induced oxidative stress. This results in the release of cytochrome C from the mitochondria, which activates caspases. Caspases are a family of cell-death proteases that play a crucial role in the execution phase of apoptosis. Ovarian damage primarily begins with an increase in the permeability of the mitochondrial membrane due to oxidative stress caused by chemotherapy, leading to the release of cytochrome C from the mitochondria, which causes the activation of caspases [30]. Therefore, targeting the apoptotic pathway could be a therapeutic strategy to protect the ovaries against cisplatin-induced damage.

Resveratrol has beneficial effects on cisplatin-induced damage by reducing mitochondrial-dependent apoptotic cell death, as evidenced by a significant decrease in the expression of both cytochrome C and caspase 3 [21]. Apoptosis is one of the mechanisms underlying hyperthermia-associated cell death. Downregulation of p53 and bcl-2 expression, as well as upregulation of Bax expression occurs [31]. In our study, we observed an increase in the expression of p53, Bax and caspases involved in the apoptotic pathway and a decrease in bcl-2 expression in all groups treated with cisplatin. Sevoflurane anesthesia alone was found to have a therapeutic effect on the apoptotic pathway, compared to cisplatin.

The results of studies on the genotoxicity and cytotoxicity of inhalation anesthetics are generally contradictory. Kvolik et al. have shown in vitro that halothane, isoflurane, and sevoflurane have cytotoxic and antiproliferative effects on tumor cells at anesthetic doses [32]. Szyfter et al. concluded that sevoflurane had no effect on peripheral blood lymphocytes [33]. It was shown that both chemotherapeutic and anesthetic drugs (cisplatin and sevoflurane) have the potential to cause DNA damage at different levels, depending on the cell type. These results indicate that sevoflurane may affect the genotoxic effects of cisplatin on healthy and tumor cells [34].

The results of Brozovic et al.’s study confirmed the genotoxic activity of anesthetics applied to EAT cells. The data indicate different capacities of different anesthetics to generate reactive oxygen species (ROS) and different genotoxic and cytotoxic effects on EAT cells. By combining with cisplatin, inhalation anesthetics may be able to inhibit necrotic or apoptotic cell death [12]. A study suggested that sevoflurane and isoflurane’s genotoxicity could lead to apoptosis in normal human cells in a dose-dependent and time-dependent manner. It argued that apoptosis is definitely associated with excessive production of ROS [35]. There are different interactions between anesthetics and anticancer agents in tumor cells, and this confirms the possibility of such interactions in healthy cells as well.

The study has some limitations, as follows: animals were studied, and there was a small sample size (research is needed using well-designed studies in humans); ketamine was also administered intraperitoneally; its interaction with HIPEC is not understood, and there is no documented malignancy in rabbits, so it cannot be assumed that, in the absence of malignancy, the results of this study should be accepted. Other limitations are that only inhalational desflurane and sevoflurane were used; anesthesia also comprises TIVA, i.e., total intravenous anesthesia with propofol, which has anti-cancer effects also. Also, the absence of the expression of the proteins and the absence of hemodynamic parameters such as arterial blood pressure or pulse could evaluated as limitations.

## 5. Conclusions

The application of cisplatin with an intraoperative HIPEC method caused ovarian damage. Sevoflurane anesthesia was found to be a better option in mitigating cell death on ovarian reserve (follicle count) and apoptosis in HIPEC procedures.

Our results need to be supported by clinical and experimental studies to elucidate the possible mechanisms underlying the interaction between anesthetic and anticancer drugs in the treatment of cancer patients. The proper selection of anesthetics for anticancer treatment may potentially enhance the effectiveness of cancer therapy and prevent possible drug interactions that cause side effects on cancer patients. Further studies with different animal models or cell lines should be conducted to confirm and expand these findings. We recommend the investigation into potential molecular pathways and mechanisms in future studies. In our study, the effect of anesthetics on cancer treatment efficacy was not discussed. We believe that it would be beneficial to investigate whether these anesthetics affect the anticancer effects of HIPEC treatment.

## Figures and Tables

**Table 1:** p53, Bax, bcl-2, and caspase 3 gene expressions and light microscopic data in the ovarian tissues of rats [Mean ± SD].

	Group C (*n* = 6)	Group H (*n* = 6)	Group HS (*n* = 6)	Group HD (*n* = 6)	*p* **
Primordial	271.17 ± 23.37	63.50 ± 12.52 *	196.00 ± 36.81 +	87.17 ± 6.64 *,&	<0.0001
Primer	75.67 ± 7.74	17.33 ± 4.07 *	60.33 ± 14.70 +	27.00 ± 2.84 *	<0.0001
Secondary	20.00 ± 5.09	5.67 ± 1.76 *	10.00 ± 2.96	14.17 ± 2.02	0.033
Tertiary	3.83 ± 0.70	3.50 ± 0.76	10.00 ± 1.91 *,+	5.50 ± 0.72	0.002
Bax	3.00 ± 0.58	51.33 ± 5.93 *	39.17 ± 1.01 *,+	47.17 ± 1.60 *	<0.0001
Bcl-2	62.17 ± 2.74	23.50 ± 4.23 *	47.00 ± 4.44 *,+	36.33 ± 1.33 *	<0.0001
Caspase 3	4.50 ± 0.76	43.50 ± 4.58 *	36.83 ± 2.74 *	40.00 ± 2.45 *	<0.0001
p53	4.00 ± 0.37	52.67 ± 5.36 *	28.00 ± 5.15 *,+	35.83 ± 3.31 *,+	<0.0001

*p* **: Significance value by One Way ANOVA test *p* < 0.05. * *p* < 0.05: compared to Group C. + *p* < 0.05: compared to Group H. & *p* < 0.05: compared to Group HS.

## Data Availability

All data generated or analyzed during this study are included in this published article.
